# *Clostridium butyricum* Combined with *Bifidobacterium infantis* Probiotic Mixture Restores Fecal Microbiota and Attenuates Systemic Inflammation in Mice with Antibiotic-Associated Diarrhea

**DOI:** 10.1155/2015/582048

**Published:** 2015-02-23

**Authors:** Zongxin Ling, Xia Liu, Yiwen Cheng, Yueqiu Luo, Li Yuan, Lanjuan Li, Charlie Xiang

**Affiliations:** ^1^Collaborative Innovation Center for Diagnosis and Treatment of Infectious Diseases, State Key Laboratory for Diagnosis and Treatment of Infectious Diseases, The First Affiliated Hospital, School of Medicine, Zhejiang University, Hangzhou, Zhejiang 310003, China; ^2^Intensive Care Unit, The First Affiliated Hospital, School of Medicine, Zhejiang University, Hangzhou, Zhejiang 310003, China

## Abstract

Antibiotic-associated diarrhea (AAD) is one of the most common complications of most types of antibiotics. Our aim was to determine the efficacy of *Clostridium butyricum*, *Bifidobacterium infantis*, and their mixture for AAD treatment in mice. AAD models were administered with single probiotic strain and probiotic mixture for short term and long term to evaluate the changes of the composition and diversity of intestinal microbiota, histopathology of the colon, and the systemic inflammation. Our data indicated that long-term probiotic therapy, but not short-term course, exerted beneficial effects on the restoration of the intestinal microbiota, the recovery of the tissue architecture, and attenuation of systemic inflammation. All predominant fecal bacteria reached normal level after the long-term probiotic mixture treatment, while IL-10, IFN-*γ*, and TNF-*α* also returned to normal level. However, the efficacy for AAD was time dependent and probiotic strain specific. Short-term administration of probiotic strains or mixture showed no apparent positive effects for AAD. In addition, the beneficial effects of *C. butyricum* combined with *B. infantis* probiotic mixture were superior to their single strain. This research showed that supplementation with *C. butyricum* combined with *B. infantis* probiotic mixture may be a simple and effective method for AAD treatment.

## 1. Introduction

Antibiotic-associated diarrhea (AAD) is the most common adverse effect of antimicrobial therapy, especially those with a relatively broad spectrum such as aminopenicillins, cephalosporins, and clindamycin [1, 2]. Generally, the mechanism by which AAD occurs most likely relates to disturbances of microbiota in the gastrointestinal tract, shifting the gastrointestinal microbiota from eubiosis to severe dysbiosis [3]. Evidence has shown that cytokines disturbance was observed after antibiotic treatment [4–6]. Approximately 1000 species of bacteria inhabit the gastrointestinal tract, and a balance of these microorganisms is crucial to normal gastrointestinal function [7]. Dysbiosis of the gastrointestinal tract may disturb the metabolism of carbohydrates, resulting in malabsorption of osmotically active particles (i.e., diarrhoea) [8]. Probiotics are live nonpathogenic microorganisms which provide a health benefit to the host when administered in adequate amounts. Previous studies have demonstrated that a variety of different types of probiotics such as* Saccharomyces boulardii*,* Lactobacillus rhamnosus* GG, and probiotic mixtures were shown as effective therapies for AAD, which can restore the altered intestinal microbiota [9]. As probiotics,* Clostridium butyricum* produces high levels of butyrate, which can decrease the intestinal permeability and reinforce various components of the colonic defense barrier such as the promotion of epithelial migration and the induction of mucins, intestinal trefoil factor, transglutaminase activity, antimicrobial peptides, and heat shock proteins [10].* Bifidobacterium infantis* exhibits a decrease in colonic permeability, an attenuation of colonic inflammation, and a decrease in interferon-gamma secretion [11]. The probiotic mixture of* C. butyricum* combined with* B. infantis* has been used to treat the observed dysbiosis in China for several years. However, it is unclear whether similar benefits occur in AAD, as most of clinical observational studies were only focused on the improvement of symptoms and signs. Using an AAD model in male C57BL/6 mice, our present study aimed to evaluate the modulation role of the probiotic mixture on the fecal microbiota and inflammatory cytokines with different courses.

## 2. Materials and Methods

### 2.1. Probiotic Strains

Two freeze-dried probiotic strains and its mixture (Changlekang) were used in present study,* Clostridium butyricum* (CGMCC 0313-1) and* Bifidobacterium infantis* (CGMCC 0313-2), which were kindly provided by Shandong Kexing Bioproducts Co., Ltd. (China). The freeze-dried* C. butyricum* powder contained viable bacteria at 5.6 × 10^9^ colony-forming units (CFU)/g and spore number at 4.4 × 10^9^ CFU/g, and* B. infantis* powder contained viable bacteria at 2.3 × 10^11^ CFU/g, while the probiotic mixture contained viable* C. butyricum* at 2.4 × 10^8^ CFU/g and viable* B. infantis* at 1.8 × 10^9^ CFU/g. Directly before administration, the probiotic products were reconstituted in sterile saline for 15 min at 37°C. The finial concentrations were 2.3 × 10^9^ CFU/mL for* C. butyricum*, 5 × 10^10^ CFU/mL for* B. infantis*, and 1.0 × 10^8^ CFU/mL* C. butyricum* and 1.0 × 10^9^ CFU/mL* B. infantis* in the probiotic mixture for further use.

### 2.2. Animals and Experimental Design

One hundred and twenty specific pathogen-free (SPF) male C57BL/6 mice (20 ± 1.3 g), purchased from the Experimental Animal Center of Zhejiang Province (Zhejiang, China), were included in our present study. AAD mice models were administered with ceftriaxone (8 g/kg body weight, Rocephin, Shanghai Roche Pharmaceuticals Ltd., Shanghai, China) intragastrically through a ball-tipped stainless steel gavage needle once daily for 5 days except the normal group [5, 6]. In order to explore the time-dependent effects of the probiotics, AAD mice models were administered with probiotics for short term (5 days) and long term (15 days). These mice were randomized into six groups of ten mice each. Besides the normal control group and AAD model groups, other four AAD groups were treated with sterile saline,* C. butyricum* (1.2 × 10^9^ CFU),* B. infantis* (2 × 10^10^ CFU), and probiotic mixture (4 × 10^7^ CFU* C. butyricum* and 4 × 10^8^ CFU* B. infantis*), respectively. The weight and stool characteristics of the mice were monitored daily. At the end of the experimental period, the mice were anesthetized by an intraperitoneal injection of 400 mg/kg body weight chloral hydrate (Sinopharm Chemical Reagent Co. Ltd., Shanghai, China) to collect their colon contents, serum, and colon tissues for further analysis. All animals were allowed to adjust to these conditions for 1 week prior to experiments, which were kept under stable housing conditions with a 12-hour light/dark cycle and free access to water and food throughout the experiment. The study protocol was approved by the Animal Care Committee of Zhejiang University, China.

### 2.3. Bacterial Genomic DNA Extraction

Frozen colon contents were thawed, and bacterial genomic DNA was extracted using QIAamp DNA Stool Mini Kit (Qiagen, Hilden, Germany) according to the manufacturer's instructions, with the additional glass-bead beating steps on a Precellys 24 homogenizer (Bertin Technologies, Montigny, France). Bacterial genomic DNA was eluted in 60 *μ*L of elution buffer and stored at −20°C for further analysis

### 2.4. PCR-DGGE Analysis

For amplification of bacterial DNA, universal bacterial primers 341F and 534R for the V3 regions of 16S rRNA genes were used and the reaction conditions were set as described by our previous studies [12, 13]. DGGE analysis of the PCR products were performed as described by Muyzer et al. [14, 15] and Ling et al. [13] with 35% to 60% gradient, using a D-Code system (Bio-Rad, Hercules, CA, USA).

### 2.5. qPCR for Fecal Predominant Bacteria

The qPCR assay was performed with a Power SYBR Green PCR Master Mix (Takara, Dalian, China) on ABI ViiA7 real-time PCR system (Applied Biosystems, Carlsbad, CA) according to the manufacturer's instructions. Bacterial specific primer sets and the reaction conditions used for qPCR were performed according to previous study [16]. The copy number of target DNA was determined by comparison with a 10-log-fold diluting standards plasmid DNA running on the same plate. Data analysis was conducted with ViiA 7 software v1.1. All reactions were carried out in triplicate repeats and a nontemplate control was performed in every analysis. Bacterial quantity was presented as log 10 bacteria per gram of feces (wet weight).

### 2.6. Histopathology

Samples from colon were fixed, paraffin-embedded, sectioned at 5 *μ*m, processed for H&E staining, and then examined under light microscopy. Tissue slides were examined in an Olympus microscope (Olympus, Tokyo, Japan).

### 2.7. Serum Cytokines Analysis

Serum interleukin-1*β* (IL-1*β*), IL-10, tumor necrosis factor *α* (TNF-*α*), and interferon *γ* were determined by commercially available mouse Raybio sandwich enzyme-linked immunosorbent assay (ELISA) kits (RayBiotech, Norcross GA, USA). Lower limit of detection for each assay was 5 pg/mL. Standard curves were generated for every plate and the average zero standard optical densities were subtracted from the rest of the standards, controls, and samples to obtain a corrected concentration.

### 2.8. Statistical Analysis

The DGGE analysis and the similarities among different bacterial DGGE profiles by Quantity One 1-D Analysis software were carried out as described previously [13]. A similarity matrix was constructed using Dice's similarity coefficient. A dendrogram was constructed by the unweighted pair group method, using arithmetic averages (UPGMA). The normally distributed continuous data are presented as the mean ± standard deviation (SD) and the differences between groups were evaluated by one-way ANOVA or Student's *t*-test. The differences between categorical data and the correlations between variables were tested by Fisher's exact test and Spearman rank correlation, respectively. All statistical analyses were performed using SPSS 18.0 (SPSS Inc, Chicago, IL) and were considered statistically significant if *P* < 0.05.

## 3. Results

### 3.1. Structural Modulation of the Fecal Predominant Microbiota by Probiotic Therapy

**Figure 1:**
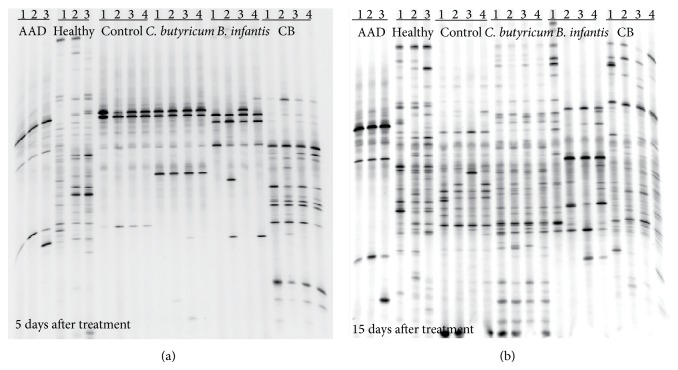
PCR-DGGE analysis of the predominant fecal microbiota in mice. PCR-DGGE fingerprints analyzed the fecal microbiota of samples from AAD mice model treated with different probiotic strains or probiotic mixture for short-term (a) or long-term (b) course. Each lane represented one subject which was selected in its group at random.

As shown in Figure 1, PCR-DGGE profiles showed the overall structure and diversity of the fecal predominant bacteria after treatment with probiotics. In the AAD mice model, most of the bacteria in the fecal microbiota have been eliminated after antibiotics treatment. After short-term probiotic therapy, the bacterial profiles were still not restored, although the numbers of bands in the probiotic mixture group were more than those in single strain groups (Figure 1(a)). When the probiotic therapy was prolonged to 15 days, there were dramatic changes in the richness and diversity of the fecal predominant bacteria in the* C. butyricum* and probiotic mixture groups, which indicated the restoration of fecal microbiota after treatment (Figure 1(b)). However,* B. infantis* could not restore the fecal microbiota successfully even after long-term administration. Cluster analysis of the DGGE profiles, which was based on the similarity indices, also demonstrated that long-term administration of probiotic mixture could restore the fecal microbiota (see Figure S1 in Supplementary Material available online at http://dx.doi.org/10.1155/2015/582048).

### 3.2. Quantification of Predominant Fecal Bacteria by qPCR

**Figure 2:**
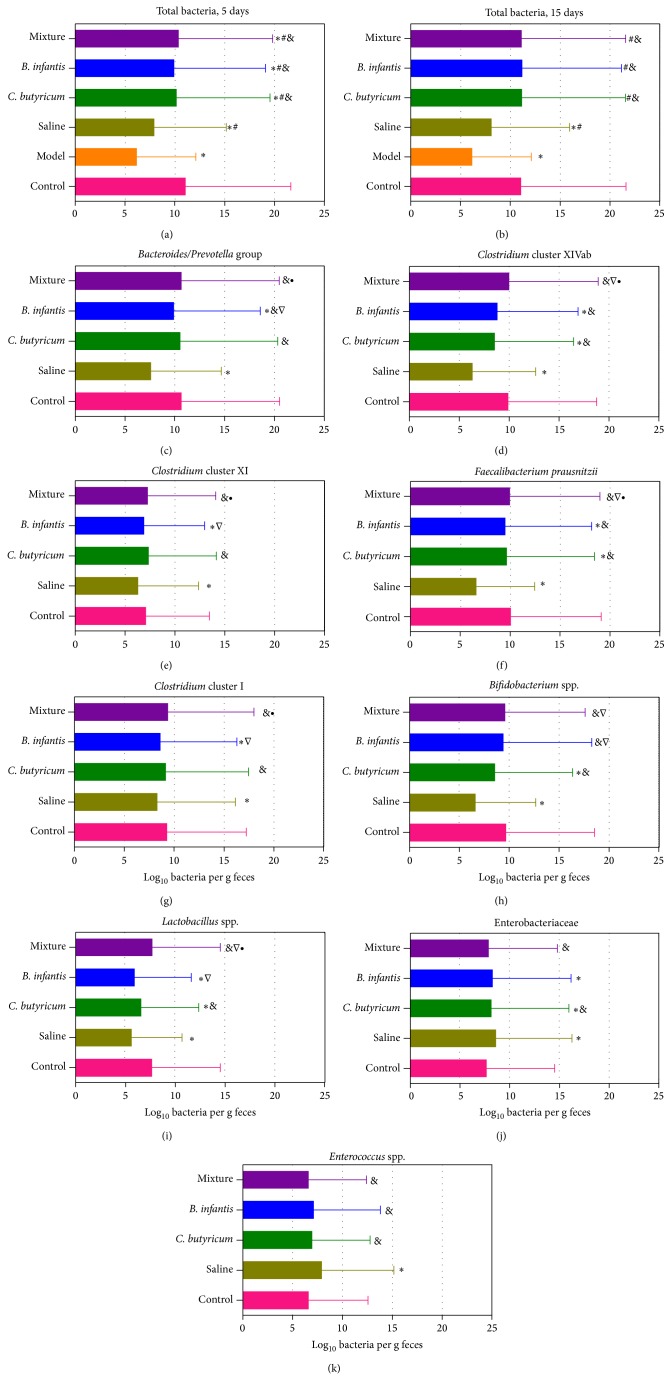
Bacterial loads in fecal microbiota as measured by qPCR (log_10_ copies/g fresh feces). The total bacteria were detected in the short-term and long-term treatment, while other predominant fecal microbiota were only detected in long-term treatment. Graph values are reported as the mean and standard deviation of the mean. Comparisons among the groups were calculated with Student's *t*-tests. *P* < 0.05 was labeled; ∗ compared with healthy control; # compared with AAD mice model; & compared with saline control; ∇ compared with* C. butyricum* treated group; ∙ compared with* B. infantis* treated group.

The differences in predominant fecal bacteria after treatment with different probiotic strains and different terms were detected by qPCR (Figure 2). After 5-day treatment, the total bacteria in these treated groups could not reach healthy levels, with approximately one order of magnitude decrease when compared with the healthy mice. However, the total bacteria returned to normal level after 15-day treatment except the saline control group, which indicated that long-term administration of probiotic strains or probiotic mixture demonstrated a positive effect on modulating the intestinal microbiota in mice. In order to explore the specific modulation of the fecal microbiota, other nine predominant bacteria were detected after 15-day treatment. Our data demonstrated that* Bacteroides*-*Prevotella* group,* Clostridium* cluster XI,* Clostridium* cluster I, and* Enterococcus* were restored into normal level in the* C. butyricum* group, while only* Bifidobacterium* and* Enterococcus* reversed in the* B. infantis* group. Intriguingly, all predominant fecal bacteria reached normal level after the probiotic mixture treatment.* Clostridium* cluster XIVab,* F. prausnitzii*,* Bifidobacterium*, and* Lactobacillus* were significantly increased in the probiotic mixture group when compared with the* C. butyricum* group, while* Bacteroides*-*Prevotella* group,* Clostridium* cluster XIVab,* Clostridium* cluster XI,* F. prausnitzii*,* Clostridium* cluster I, and* Lactobacillus* were obviously higher in the probiotic mixture group than that in the* B. infantis* group. These observations suggested that the combined* C. butyricum* with* B. infantis* could restore the fecal microbiota more efficiently than the single probiotic strain.

### 3.3. Histopathology of the Colon

**Figure 3:**
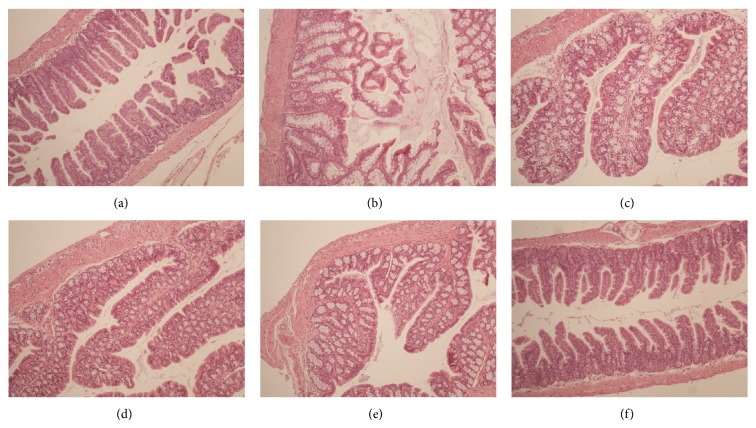
Histopathology of the colon after treatment. Images represent sections of the distal colon (magnification, ×40). (a) Healthy control; (b) AAD mice model; (c) saline control; (d)* C. butyricum* treated group; (e)* B. infantis* treated group; (f) probiotic mixture treated group.

The evident damage of colon architecture after antibiotic treatment was loss of homogenously distributed and integrated villi (Figure 3). Most of the colon epithelial cells showed severe swelling and partially rupturing. After 15-day actively treatment, the tissue architecture of the colon was restored significantly in the* C. butyricum* combined with* B. infantis* group, with the villi homogenously distributing as the healthy control. However, the severe villous swelling and extending could still not return to normal level in the* C. butyricum* group and the* B. infantis* group. Our data suggested that the* C. butyricum* combined with* B. infantis* could help to restore the colon mucosa successfully in a relative long-term course.

### 3.4. Comparison of Serum Cytokines after Probiotic Treatment

**Figure 4:**
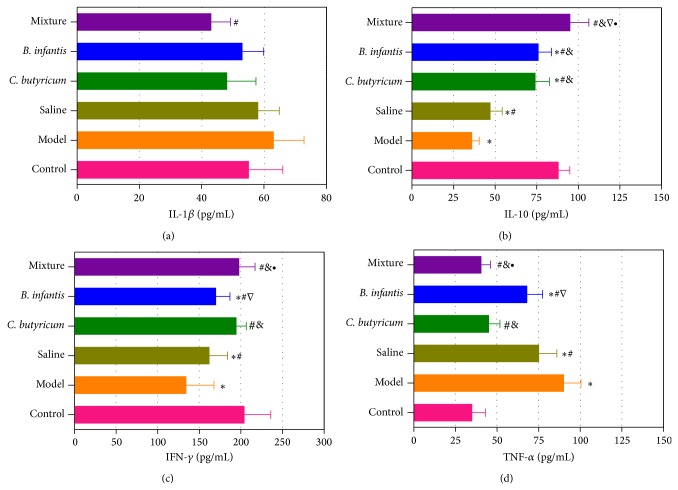
Comparison of cytokines among the six groups. *P* < 0.05 was labeled. ∗ compared with healthy control; # compared with AAD mice model; & compared with saline control; ∇ compared with* C. butyricum* treated group; ∙ compared with* B. infantis* treated group.

In the AAD mice model, the TNF-*α* level increased and the IL-10 and IFN-*γ* levels decreased significantly, which might be involved in the systemic inflammation of the mice (Figure 4). However, IL-1*β* was not significantly altered in the AAD mice. The serum levels of IL-10 and IFN-*γ* increased significantly after long-term* C. butyricum* administration, while the TNF-*α* decreased obviously, which were also observed in the* B. infantis* group (*P* < 0.05). However, those altered serum IL-10, IFN-*γ*, and TNF-*α* were still not reaching healthy levels in the* B. infantis* group, whereas only IL-10 was still lower in the* C. butyricum* group than that in healthy mice. It was unexpected that the concentration of IL-10, IFN-*γ*, and TNF-*α* returned to normal levels in the probiotic mixture group (*P* > 0.05). Our results indicated that the* C. butyricum* combined with* B. infantis* could attenuate systemic inflammation in the AAD mice.

## 4. Discussion

The emergence of variety of antibiotics has been used extensively in human and veterinary medicine, for the purpose of preventing (prophylaxis) or treating microbial infections since 1928. However, antibiotic therapy has been widely overused and misused during the past decade despite considerable evidence that antibiotic treatment of microbial infections shortens its course or prevents the development of secondary bacterial infections. In fact, widespread use of broad-spectrum antibiotics can affect not only the target pathogen but also commensal inhabitants of the human host. Normal commensal bacteria play vital roles in maintaining host-microbe homeostasis and inhibiting pathogen colonization and overgrowth (i.e., colonization resistance). These processes are attributable to the existence of a stable and diverse population of resident microorganisms that compete with an invading pathogen directly for niches and nutrients or through production of antibacterial substances [17, 18]. As a common complication of most types of antibiotics, AAD affects variety of populations including outpatients, hospitalized patients, and residents of long-term care facilities and results in extended hospital stays, increased medical care costs, and increased diagnostic procedures [1, 19]. Beniwal et al. have considered that AAD pathogenesis may be related to altered short-chain fatty acids in the intestine, functional disturbance of carbohydrate, and bile acid metabolism due to alteration of the microbiota or toxic effects on the intestinal mucosa and pharmacological effect on motility [3, 20]. So far, there are no other current effective preventive measures for AAD, except for discontinuing the inciting antibiotic or switching to an antibiotic with a narrower spectrum of action.

Increasing evidence has shown that probiotics are a promising strategy for the prevention and treatment of AAD, which can help to restore intestinal microbiota and reestablish intestinal homeostasis [9, 21]. Probiotics are living microbes taken to provide a health benefit on the host, which offer promise for a wide diversity of diseases and have an excellent safety-benefit ratio. However, the efficacy is probiotic strain specific.* C. butyricum* (*Clostridium* cluster I) is a typical butyric-acid producing gram-positive anaerobe found in the intestines of healthy human, which has been used for modulating gastrointestinal microbiota and treating intestinal disorders [22–24].* C. butyricum* can produce endospores, which is a key characteristic related to its ability to survive at lower pH, at relatively higher bile concentrations, and in the presence of coadministered antibiotics [23, 25].* B. infantis* is a normal component of the intestinal microbiota in humans and animals and is frequently associated with health-promoting effects. As a lactic acid bacterium,* B. infantis* has been proved to be useful probiotics that can specifically relieve many of the symptoms of irritable bowel syndrome [26]. Previous studies also demonstrated that* C. butyricum* promotes the growth of* Lactobacillus* and* Bifidobacterium* and inhibits AAD in human and mice [23, 27, 28]. In our present study, two probiotic strains and their mixtures mentioned above were used to treat the AAD. In the AAD mice model, the intestinal microbiota was almost eliminated as only few bands in the DGGE profiles, and the tissue architecture of the colon was damaged dramatically. Regardless of the short-term or long-term follow-up time, the intestinal microbiota and the tissue architecture of the colon could still not recover naturally. Our data demonstrated that the probiotic strains and mixtures exerted beneficial effects on the restoration of the intestinal microbiota and the recovery of the tissue architecture. However, the effects were time dependent and strain specific. Short-term treatment had little influence on the composition and diversity of the intestinal microbiota. In addition, the probiotic* C. butyricum* was more effective than* B. infantis*, even though high dose of* B. infantis* was used. The discrepancy of the therapeutic effects might be associated with their different metabolites, especially short-chain fatty acids [29]. These metabolites might improve the ecosystem of the gastrointestinal tract via promoting the growth of healthy symbionts and/or enhancing the barrier function of epithelial cells [30]. Wong et al. have shown that butyrate, rather than lactic acid, plays a central role in maintaining gut homeostasis [31]. However, it was apparent that the* C. butyricum* combined with* B. infantis* probiotic mixture was superior to their single probiotic strain in the treatment of AAD. The predominant bacteria and the tissue architecture that were broken down by antibiotics were almost restored after a relative long-term probiotic mixture treatment. The advantage of using this probiotic mixture containing* C. butyricum* and* B. infantis* may derive from the synergistic effects of the two bacteria.

In addition, the development of AAD was also accompanied with systemic inflammation as the proinflammatory cytokines increased and anti-inflammatory cytokines decreased significantly. In present study, the probiotic strains and probiotic mixture showed immunomodulatory effects in the AAD mice. The increase of TNF-*α* and decreases of IL-10 and IFN-*γ* in the AAD mice were significantly recovered in all probiotic groups. Previous studies have demonstrated that* C. butyricum* could suppress intestinal immune disorders by regulating IL-10 production [32], while* B. infantis* could attenuate inflammation in DSS-induced colitis in rats [33]. Our study also indicated that the immunomodulatory effects of the probiotic mixture were superior to the single strain of* C. butyricum* and* B. infantis*. These cytokines seemed to be involved in the beneficial effects of probiotics on AAD; however, the mechanisms by which the probiotic mixture modulated immune function were still unclear. Future researches on the mechanisms of action of the probiotic mixture in vitro are required.

## 5. Conclusion

In present study, we found that long-term administration of* C. butyricum* combined with* B. infantis* probiotic mixture exerted beneficial effects on the restoration of the intestinal microbiota and the recovery of the tissue architecture of the colon, which was superior to their single probiotic strain in the treatment of AAD. Moreover, the possible protective role might be associated with the immunomodulatory effects of the probiotic mixture. According to our present results, supplementation with* C. butyricum* combined with* B. infantis* probiotic mixture is a simple and effective method to treat AAD.

## Supplementary Material

Figure S1: UPGMA dendrogram of the DGGE profiles for short-term (A) and long-term (B) treatment. Based on the similarity indices, our present cluster analysis of the DGGE profiles demonstrated that long-term administration of probiotic mixture could restore the fecal microbiota.
