# Supplementary figures and images for: Clostridium butyricum Combined with Bifidobacterium infantis Probiotic Mixture Restores Fecal Microbiota and Attenuates Systemic Inflammation in Mice with Antibiotic-Associated Diarrhea
Source: Biomed Res Int. 2015 Feb 23;2015:582048. doi: 10.1155/2015/582048 (PMC4352745; doi:10.1155/2015/582048)

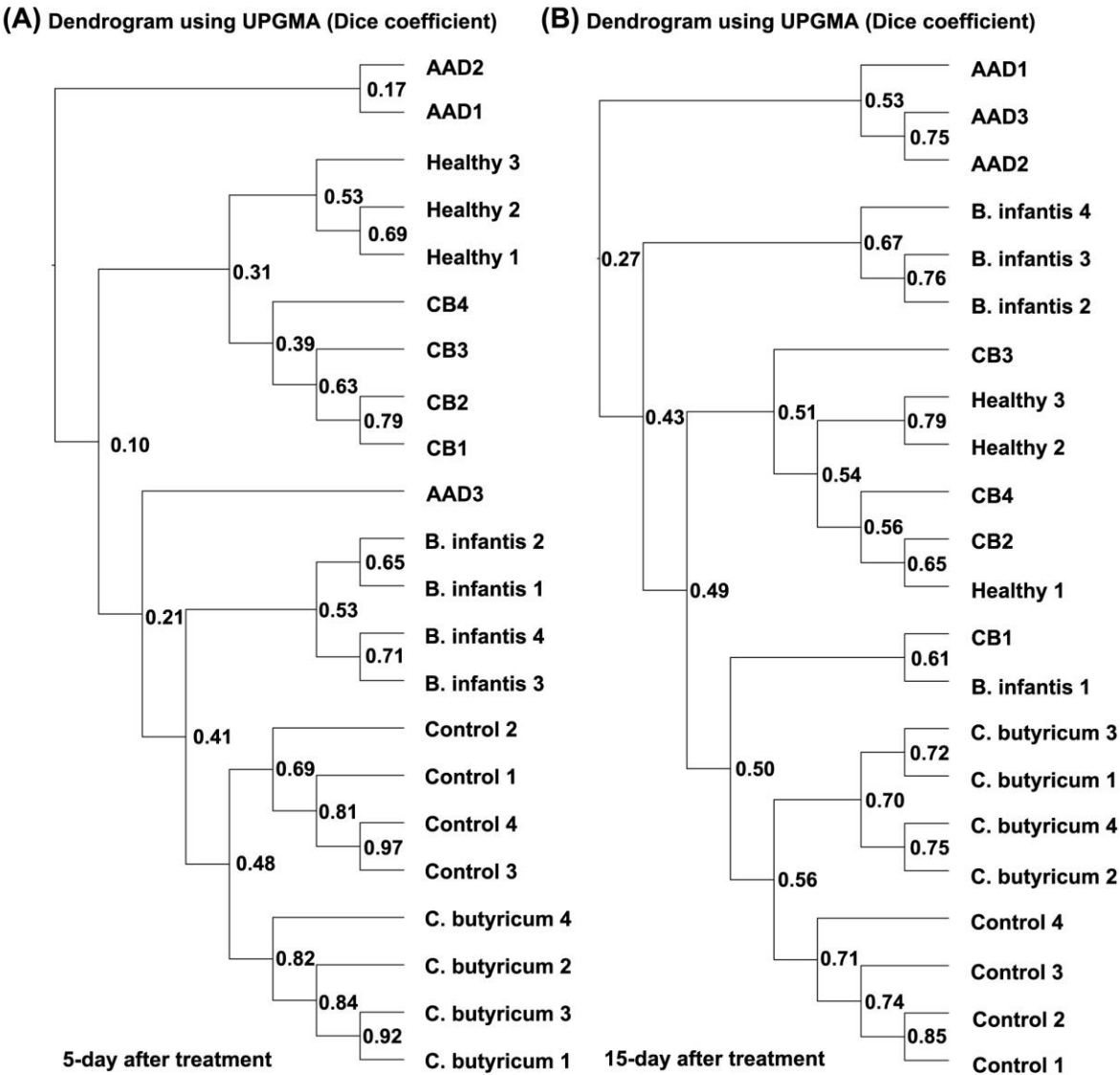

Supplement: Supplementary file 1 — Figure S1: UPGMA dendrogram of the DGGE profiles for short-term (A) and long-term (B) treatment. Based on the similarity indices, our present cluster analysis of the DGGE profiles demonstrated that long-term administration of probiotic mixture could restore the fecal microbiota. [file 582048.f1.pdf]
